# Sandy Lake Health and Diabetes Project: A Community-Based Intervention Targeting Type 2 Diabetes and Its Risk Factors in a First Nations Community

**DOI:** 10.3389/fendo.2013.00170

**Published:** 2013-11-12

**Authors:** Kara E. Kakekagumick, Mariam Naqshbandi Hayward, Stewart B. Harris, Brit Saksvig, Joel Gittelsohn, Gary Manokeesic, Starsky Goodman, Anthony J. Hanley

**Affiliations:** ^1^Centre for Studies in Family Medicine, Department of Family Medicine, Schulich School of Medicine and Dentistry, The University of Western Ontario, London, ON, Canada; ^2^Department of Epidemiology and Biostatistics, University of Maryland School of Public Health, College Park, MD, USA; ^3^Center for Human Nutrition and Johns Hopkins Global Center of Childhood Obesity, Department of International Health, John Hopkins Bloomberg School of Public Health, Baltimore, MD, USA; ^4^Sandy Lake Health and Diabetes Program, Sandy Lake First Nation, ON, Canada; ^5^Departments of Nutritional Sciences and Medicine and Dalla Lana School of Public Health, Faculty of Medicine, University of Toronto, Toronto, ON, Canada

**Keywords:** diabetes mellitus, community-based intervention, aboriginal, first nations, participatory research, prevention, epidemiology

## Abstract

The Sandy Lake Health and Diabetes Project (SLHDP) was initiated in 1991 as a partnership between Sandy Lake First Nation and researchers interested in addressing the high rates of type 2 diabetes mellitus (T2DM) in the community. Following the expressed wishes of the community, the SLHDP has encompassed a variety of community-wide interventions and activities including: community surveys to document T2DM prevalence and risk factors, the Northern Store program aimed at increasing the availability and knowledge of healthy food options, a home visit program for the prevention and management of T2DM, a local diabetes radio show, a school diabetes curriculum for grades 3 and 4, a community-wide walking trail to encourage increased physical activity, youth diabetes summer camps, and a variety of community events focusing on nutrition and physical activity. Over the 22 year existence of the SLHDP, the community has taken ownership of the program and activities have evolved in alignment with community needs and priorities. This paper discusses the history, implementation, evaluation, and outcomes of the SLHDP and describes its sustainability. The SLHDP is a model of culturally appropriate participatory research that is iterative, with reciprocal capacity building for both key community stakeholders and academic partners.

## Introduction

Type 2 diabetes mellitus (T2DM) in First Nations communities in Canada is a significant clinical and public health problem (1, 2). The prevalence and incidence of T2DM have been increasing rapidly over the past several decades, and the condition is a major cause of morbidity and mortality in this population (1). This increase in the T2DM disease burden can be attributed in part to drastic lifestyle changes in the last 50 years, with a sharp decrease in physical activity and a marked nutritional transition from traditional to more Western diets (3). Community-based interventions are an optimal approach to address the diabetes burden as they allow communities to participate fully as partners in the development and implementation of primary and secondary prevention initiatives (4, 5). In addition, and in contrast to targeted, individual-level interventions, community-based approaches are able to reach a larger proportion and broader spectrum of the population (6, 7). Community-based interventions have been implemented in Sandy Lake First Nation (SLFN) in Northwestern Ontario (8–10) and Kahnawake First Nation in Quebec (11); these are both model programs for other First Nations communities struggling with diabetes (12–16).

This paper discusses the history, development, implementation, evaluation, and sustainability of the community-based intervention program in SLFN. The community-based intervention program has been a central and long-standing component of the Sandy Lake Health and Diabetes Project (SLHDP) (8, 9). The key objectives of this program have been to work with community organizations and individuals to develop strategies to educate community members regarding diabetes prevention and management, and to improve environments for making healthy lifestyle choices, especially in the context of physical activity and nutrition (9). Evaluation of the program has been carried out through both qualitative and quantitative research methods. Sustainability of SLHDP can be attributed to the community ownership and participation in programing.

### Community setting: Sandy Lake First Nation

Sandy Lake First Nation is an Ojibway–Cree community located in the subarctic boreal forest region in central Canada. The total registered population of Sandy Lake as of June 2013 was estimated to be 2866 people (17). It is a remote fly-in community only accessible by air for most of the year (18). SLFN has a Northern Store, which is the main grocery store and the only location to purchase fresh produce in the community, as well as four other small locally owned businesses that sell food and other goods (18). The community has two schools for students from kindergarten to grade 10. To graduate high school, students must leave the community to enroll in schools in southern communities, or complete their diploma requirements via distance education. Healthcare in SLFN is delivered from a federally operated Nursing Station (18). The community also has a dedicated Diabetes Prevention Program, which is separate from the clinical care and management services offered by the Nursing Station. Two community members lead the diabetes prevention [GM and SG], which aims to decrease the prevalence of diabetes in SLFN by encouraging exercise and proper nutrition through a variety of community programing activities. This program is recognized as critically important by both the community and the researchers, as the 10-year cumulative incidence of type 2 diabetes in a recent prospective study was 17.5% (19). There is also a heavy burden of risk factors for diabetes; for example, the overall prevalence of overweight in children aged 2–19 years was 27.7% in boys and 33.7% in girls (20).

Traditionally, the people of SLFN were hunter-gatherers and led a physically demanding lifestyle (18). Their diets consisted of animal protein from wild game and fish, as well as roots and seasonal berries (18). Their traditional lifestyle eroded with the development of the Indian reserve and residential school systems. The shift from a high protein diet to one high in refined carbohydrates and saturated and trans fat has been detrimental to the health of Sandy Lake and other First Nations communities in Canada. This has caused an increase in the prevalence of diabetes and associated comorbidities (1, 2, 21, 22).

Similar to many remote First Nations communities in Canada, SLFN continues to face challenges in its health promotion efforts focused on diabetes. More specifically, accessibility to, and costs of, healthy foods are major concerns, given the high prices and the difficulty of transporting fresh and perishable foods to the local Northern grocery store. Despite recent advances with community-based activities geared toward increasing physical activity in SLFN, inadequate environmental and social support for physical activity remains an on-going challenge. SLFN faces long winters and a lack of adequate indoor facilities, which contributes to a poor environment for physical activity.

In 1991, the Chief and Council of SLFN approached Dr. Stewart Harris, the medical director of Sioux Lookout Zone at the time, to voice their concern regarding the increasing prevalence of diabetes in the community. This meeting led to further discussions and eventually an agreement between the community and Dr. Harris to initiate the SLHDP.

## Sandy Lake Health and Diabetes Project

The overarching objectives of the SLHDP were to:

1. Determine the prevalence of diabetes and impaired glucose tolerance (IGT) in Sandy Lake using the gold-standard oral glucose tolerance test approach.
2. Identify anthropometric, metabolic, and lifestyle characteristics associated with diabetes and IGT in the community.
3. Use ethnographic data collection techniques to aid in the development of culturally appropriate intervention strategies to modify risk factors for diabetes and its complications, with a specific emphasis on improving diet and increasing physical activity.
4. Provide a model intervention strategy that could be implemented in other First Nations communities across Ontario (8, 9).

**Figure 1:**
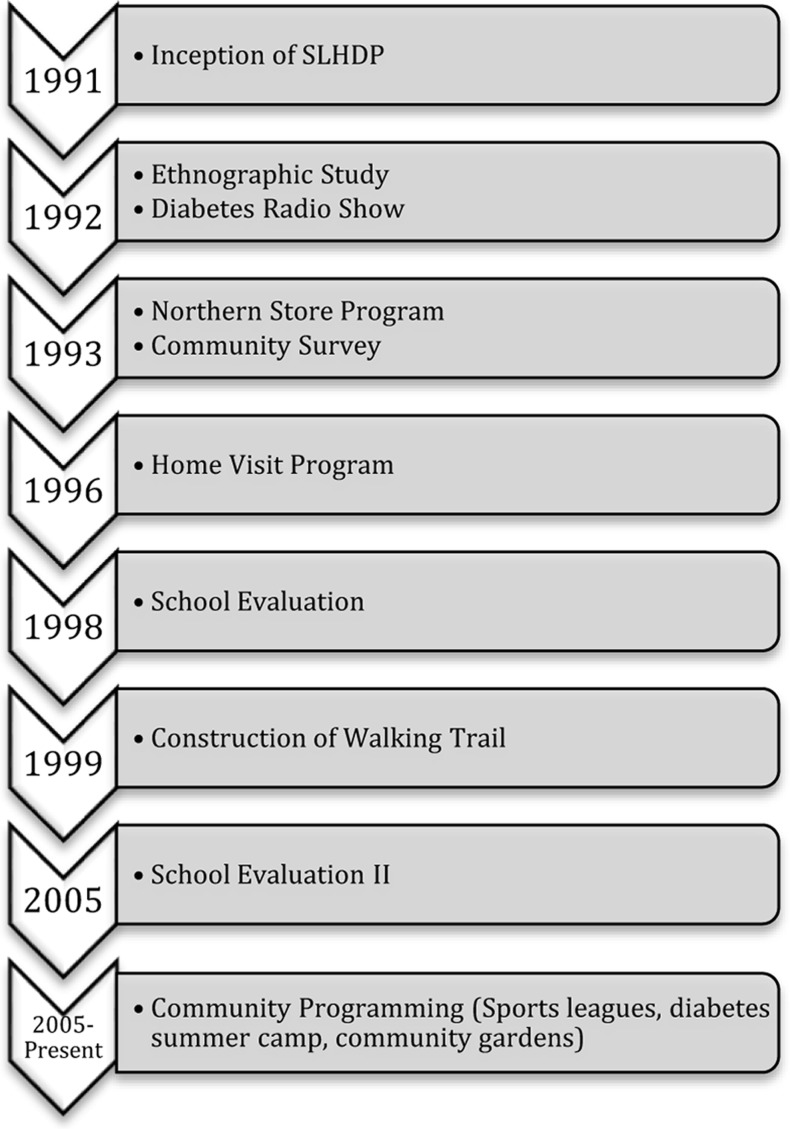
**Progression of the SLHDP from 1991 to present**.

The purpose of the present paper is to describe the development, succession, and evaluation of the SLHDP intervention strategies in the context of community engagement process. Figure 1 shows the progression of the SLHDP from 1991 to the present.

Over the years, by employing formative and feasibility research and pilot testing, successful strategies have emerged. Intervention elements included a Northern store program, home visit program, a diabetes radio show, and a school-based diabetes curriculum, all of which encouraged engagement in physical activity and the promotion of healthy lifestyles (8, 9). The program has received community support, including participation from the community and partnerships with other programs and organizations in the community. A critically important feature of the SLHDP intervention is that the programs to encourage healthy eating and physical activity are culturally appropriate and have been adapted to suit the needs of the community through a participatory research approach (8).

### Formative research studies: 1991–1996

The Sandy Lake Health and Diabetes Project was initiated in 1991 with initial formative studies focused on qualitative research (8). The ethnographic study collected information on health beliefs and attitudes, perceptions of food and physical activity, notions of disease causation and opinions on determinants of health, disease prevention, and body image concepts (8, 23, 24). The goals of this initial qualitative and ethnographic work were to gain community perspectives on diabetes and its risk factors in order to determine potential strategies for addressing the diabetes problem. This data formed the basis of SLHDP’s community-based intervention by aiding in the development of locally appropriate strategies to modify risk factors for diabetes.

### Community survey: 1993–1995

A baseline prevalence and risk factor survey was conducted from 1993 to 1995, during which all community members age 10 and older were invited to participate in oral glucose tolerance tests (OGTTs), anthropometric measurements, risk factor questionnaires, and fitness testing (9). The study documented very high age-standardized rates of T2DM (26.1%) and IGT (13.6%) (21), and the findings received national attention among politicians and the media. The study also found that measures of obesity and fasting insulin levels were significantly associated with T2DM in the 18–49 age group (21).

Subsequently, based on the results of these initial research components, research from other sectors, local knowledge, and community feedback on pilot initiatives, a community-based intervention strategy was developed (8, 9).

### Northern food store program

In 1993, the SLHDP initiated the Northern Store program (8). This program increased the selection of low-fat foods and sugar free alternatives in the grocery store. SLHDP worked with a nutritionist at the Northern Store head office to develop labels for healthy food choices which were printed in both English and Oji-Cree syllabics, in addition to setting up information displays and taste tests to promote healthy eating. Store tours were also provided so that community members could develop label-reading skills in order to make healthy choices in the grocery store. SLHDP continues to partner with the Northern store to hold healthy food demonstrations in the store.

### Home visit program: 1996–1997

The home visit program was one of the original programs implemented by SLHDP from 1996 to 1997 to aide in health promotion and diabetes education in Sandy Lake (25). This program brought individualized teaching on nutrition, health, and physical activity directly to the homes of interested families. There were five visits to each family and each visit focused on a specific topic, with activities including cooking demonstrations, taste tests, printed educational materials, and a human physiology teaching kit which was used to describe basic human physiology as it related to diabetes. During the visits, families were shown how to prepare everyday foods using healthier cooking methods, including demonstrations of bannock recipes with whole-wheat flour and methods to drain fat from ground beef. There were 115 participants in the home visit program evaluation. Height, weight, and percentage of body fat and lean body mass were measured. Also, questionnaires were conducted at three time points: before the program, after the program, and at the 6 month follow-up point. Questionnaires determined changes in knowledge, attitudes, practices, and stages of change.

The home visit program was ultimately not sustainable and therefore, discontinued after a 1-year trial period. When the pilot program began, funding was limited and the home visits were labor intensive for the SLHDP team. The program was also not as popular as other programs that were being piloted concurrently.

### Diabetes radio show

Among the most long-standing and sustainable components has been the community diabetes radio show. Sandy Lake uses its local radio station as a means of communication as it is common for homes and workplaces to have the radio on throughout the day. The diabetes radio show airs once a week for an afternoon and provides information on diabetes prevention and management and allows community members to call in and ask questions and participate in games.

### The sandy lake school-based diabetes curriculum

During the development of the SLHDP, the Sandy Lake Chief, and Council communicated that they wanted the intervention programs to have a strong emphasis on the children. Consequently, the school-based intervention was designed to target Sandy Lake youth with the goal of improving the health of future generations. The objective of the school-based diabetes prevention program was to provide early education toward the development of lifelong skills in healthy eating and exercise to prevent the onset of diabetes. The study is described in detail by Saksvig et al. (10).

In brief, the school-based diabetes prevention curriculum was developed by a PhD student and local Oji-Cree teacher, with guidance and input from elders of the community (10). The joint effort between the SLHDP staff and the community resulted in a culturally appropriate curriculum for Sandy Lake students. The curriculum incorporated modifications of the curriculum already developed by Kahnawake Mohawk community in Quebec and the CATCH curriculum (11, 26). The third and forth grade curriculum consists of two lessons a week for 17 weeks. The lessons incorporate taste tests, skill building, goal setting, intergenerational learning, humor, games, and storytelling using Aboriginal characters. One unique adaptation of the lessons was to use storytelling as a way to explain important concepts. The stories are based on the characters Missy and Buddy Daaybway as they learn the importance of a having a healthy lifestyle to prevent diabetes.

The school curriculum intervention focused on five main components: curriculum, family, and peer involvement, environment, and a healthy meal provided at school (10). The Sandy Lake school-based diabetes curriculum was the main component of the school intervention. The family component was designed to inform parents and family about what the children were learning in school, through radio, parent-teacher nights, and letters sent home. This allowed students to apply at home what they had learned at school. The peer component encouraged students to act as role models; this was achieved by creating children’s cooking show videos and by airing a Diabetes Kids radio show. The environmental component focused on providing students with a supportive environment to make healthy food choices by providing a healthy breakfast snack to students from kindergarten to grade 5 consisting of fruit, milk, cheese, and rice cakes. Moreover, a school wide ban on high-fat and high-sugar foods, including pop and chips, was initiated by the Education Authority. Local stores were also encouraged to feature healthy snacks on their food displays to make it easier for children to make healthy food purchases (10). All students in grades 3 and 4 received the diabetes curriculum but only students with parental consent and student assent were included in the evaluations.

### School program evaluation I: 1998–1999

The first evaluation of the diabetes curriculum took place from 1998 to 1999. The goal of the evaluation was to demonstrate that after 1 year, a culturally appropriate school-based intervention would increase the students’ knowledge, skills, and self-efficacy and positively change behaviors related to diet and physical activity (10). Under the study protocol, students completed four measurements consisting of anthropometry, 24 h dietary recall, and two questionnaires at baseline and follow-up (10).

Of the 138 eligible participants, 122 (88%) completed both the baseline and the follow-up measures (10). Results showed that there were significant increases in dietary intention, dietary preference, knowledge of curriculum concepts, and dietary self-efficacy, and a decrease in screen time (Table 1). There was an increase in dietary fiber and no significant decrease in dietary fat, despite an increase in knowledge about foods that were low in dietary fat (10).

**Table 1:** **Knowledge and psychosocial factors results of the 1-year pilot of the Sandy Lake School-based Diabetes Curriculum intervention [adapted from Saksvig et al. (10)]**.

	Range	Baseline	Follow-up
Dietary fat knowledge	0–10	5.3 ± 2.3	7.1 ± 2.5***
Dietary intent	0–6	3.5 ± 1.5	4.2 ± 1.6***
Dietary preference	0–6	2.5 ± 1.5	3.2 ± 1.7 ***
Dietary self-efficacy	0–27	17.5 ± 5.4	19.6 ± 5.4**
Curriculum knowledge	0–8	2.9 ± 2.3	4.5 ± 2.4***
**SCREEN TIME**			
Dietary fiber intake	–	11.6 ± 8.0	13.4 ± 8.0
Dietary fat intake	–	86.4 ± 51.4	83.3 ± 45.1

Values are means ± SD, *n* = 122. Asterisks indicate a difference from baseline: **p < 0.01, ***p < 0.001. All tests are paired *t* tests. Change is Follow-up score – baseline score.

### School evaluation II: 2005–2006

Following the 1998 school-based intervention activities, the curriculum was taught sparingly in the schools. In 2005 researchers and community leaders implemented the curriculum again with the goal of increasing the sustainability of the program. The evaluation was modified to include a more objective measure of physical activity and four data collection periods using a pre-post study design. The second round of evaluation of the school program took place in 2005–2006 (27). Data were collected at four data collection points: September 2005, April 2006, September 2006, and April 2007. Data collection occurred before curriculum implementation in each of the September semesters and within 1–2 weeks after the last lesson in the curriculum in each of the April semesters. Physical activity was emphasized in a more targeted way in this phase of the curriculum evaluation with dedicated time for physical education and 20 min activity breaks throughout the school day. Teachers were encouraged to incorporate daily walks into their school day schedules. The evaluation consisted of a student questionnaire, 24 h dietary recall, anthropometric data, and physical activity testing. The student questionnaire consisted of demographics; dietary self-efficacy; health, nutrition, and diabetes knowledge, while a separate logbook was used to record TV/video time. Nutritional data were collected using one-to-one interviews with students where they were asked to recall their food and beverage consumption in the previous 24 h. Anthropometric data consisted of height, weight, waist circumference, body mass index (BMI), and body fat percentage and were collected at each of the four data collection points. BMI and body fat percentage were calculated using the TBF-305 Body Fat analyzer (Tanita Corp. Inc, Tokyo, Japan). The physical activity test was a 20 m shuttle test that required students to run between two cones placed 20 m apart at the same time a sound was emitted with each student’s score reflecting the highest level they could achieve prior to no longer being able to follow the pace set by the emitted beeps (28). Scores were used to calculate maximum oxygen uptake (VO_2_ max) using the formula derived by Léger et al. (28): VO_2_ = 31.025 + 3.238*X* − 3.248*A* + 0.1536*AX*, where *X* = speed (km h^−1^) calculated as (8 + 0.5 last stage no. recorded on shuttle test) and *A* = age (year) (28). Results showed improvements in self-efficacy, knowledge of health and nutrition, and screen time (27) (Table 2). Improvements did not transfer to measures of VO_2_ max and anthropometric measurements (27) (Table 2). There was also an increase in consumption of milk and grains (Table 2). A decrease in the percentage of energy from sugar was noted where approximately 30% of energy was from sugar in 2005 with a decrease to below 25% in 2006 (27).

**Table 2:** **Student demographics, anthropometric, physical activity, questionnaire TV/video game results, and nutritional results (26)**.

Variable	Data collection period				*p*-Value
	Fall 2005	Spring 2006	Fall 2006	Spring 2007	
**ANTHROPOMETRIC DATA**					
Participation rate % (*n*)	97.7 (43/44)	93.6 (44/47)	85.1 (40/47)	89.4 (42/47)	–
Retention rate (%)	81.4	79.5	87.5	83.3	–
Height (cm); mean (*n*)	136.0 (35)	139.2 (35)	141.6 (35)	145.7 (35)	<0.001
Weight (kg); mean (*n*)	38.2 (35)	41.6 (35)	44.1 (35)	49.2 (35)	<0.001
BMI; mean (*n*)	20.4 (35)	21.2 (35)	21.7 (35)	23.1 (35)	<0.001
BMI *z*-score; mean (*n*)	1.0 (35)	1.1 (35)	1.2 (35)	1.3 (35)	0.001
Waist circumference (cm); mean (*n*)	74.8 (35)	76.9 (35)	76.8 (35)	84.1 (35)	<0.001
% Body fat; mean (*n*)	32.0 (35)	33.2 (35)	33.9 (35)	35.8 (35)	<0.001
**PHYSICAL ACTIVITY**					
Participation rate % (*n*)	86.4 (38/44)	93.6 (44/47)	78.7 (37/47)	87.2 (41/47)	–
Retention rate (%)	71.0	61.4	73.0	65.8	–
VO_2_ max (ml kg^−^1 min^−^1); mean (*n*)	34.4 (27)	32.2 (27)	32.6 (27)	31.0 (27)	<0.001
**TV/VIDEO LOGS**					
Participation rate % (*n*)	97.7 (43/44)	93.6 (44/47)	87.2 (41/47)	85.1 (40/47)	–
Retention rate (%)	79.1	77.3	82.9	85.0	–
Time watching TV/playing video games (min); mean (*n*)	192.6 (34)	164.7 (34)	108.9 (34)	130.2 (34)	<0.05
**QUESTIONNAIRES**					
Participation rate % (*n*)	97.7 (43/44)	91.5 (43/47)	89.4 (42/47)	89.4 (42/47)	–
Retention rate (%)	81.4	81.4	83.3	83.3	–
Self-efficacy score^a^; mean (*n*)	2.9 (35)	3.1 (35)	3.3 (35)	3.3 (35)	0.001
Self-reported TV/video game habits^b^; mean (*n*)	2.9 (35)	3.0 (35)	3.4 (35)	3.2 (35)	<0.05
Health and dietary knowledge^c^; mean (*n*)	0.41^d^ (34)	0.57 (34)	0.65^e^ (34)	0.62 (34)	<0.001
**NUTRITIONAL RESULTS**					
Milk; mean (SE)	0.3 (0.1)^f^	0.8 (0.2)^g^	1.1 (0.2)^g^	0.7 (0.1)^g^	–
Grains; mean (SE)	1.2 (0.2)^f^	2.4 (0.5)^f,g^	4.4 (1.5)^f,g^	3.4 (0.8)^g^	–

**n* = total number of students included in the analysis, BMI = body mass index, VO_2_max = maximum volume of oxygen*.

*^a^ Self-efficacy scores ranged from 1 = low self-efficacy to 4 = high self-efficacy*.

*^b^ Self-reported habits ranged from 1 = more time reported on TV/Video to 4.5 = less time reported on TV/Video*.

*^c^ Health and dietary knowledge scores were coded as 0 = lesser knowledge, 1 = greater knowledge*.

*^d^ 93.2% (41/44) of students completed this section of the questionnaires*.

*^e^ 91.5% (43/47) of students completed this section of the questionnaires*.

*^f,g^ Means with different superscript letters are significantly different by post hoc multiple comparison using the least significant difference (LSD) pairwise multiple comparison*.

Over the years, physical activity has received greater emphasis and the diabetes prevention program staff continue to expand the program by adding more culturally appropriate and locally sustainable activities such as running clubs, sports tournaments, and skating on outdoor rinks. To encourage physical activity, there are after-school programs available to children, including sports such as baseball, hockey, and broomball.

### Community-wide walking trail

**Figure 2:**
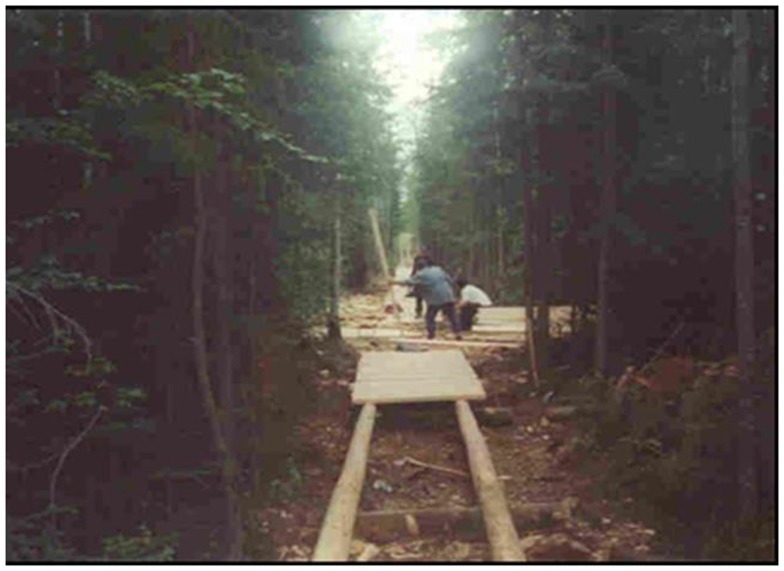
**Image showing a boardwalk portion of the walking trail under construction**.

In an effort to improve environments for physical activity, a system of walking trails (Figure 2) was initiated and developed within the community jointly by the Diabetes Prevention Program, Band Council, the community corporation, the welfare office, and the National Child Benefit program. The walking trails are 6 km long and are located away from the major roads, which do not have sidewalks and are thus not optimal for walking due to mud, dust, and traffic. The trails give the community members an improved setting for leisure walks in addition to more structured activity programs, and also provide a walking shortcut between different areas in the community. The walking trail is used for Poker Walks, which are popular community-wide competitions held several times a year to encourage people to be active. During a poker walk, the participants must walk to separate locations to pick up a random playing card, and the person with the best hand at the last station wins a prize. The trails are also used for walking and running clubs that are open to the community for participation.

### Youth diabetes summer camp

For many years, Sandy Lake has organized a diabetes summer camp for its youth. More recently, Sandy Lake has partnered with the organization Let’s Talk Science to teach the roles that nutrition and physical activity have in diabetes prevention, as well as underlying scientific concepts. The camp runs for a week in August and there are days devoted to nutrition, exercise, and traditional ecological knowledge.

### Diabetes prevention program – health promotion in the community

**Figure 3:**
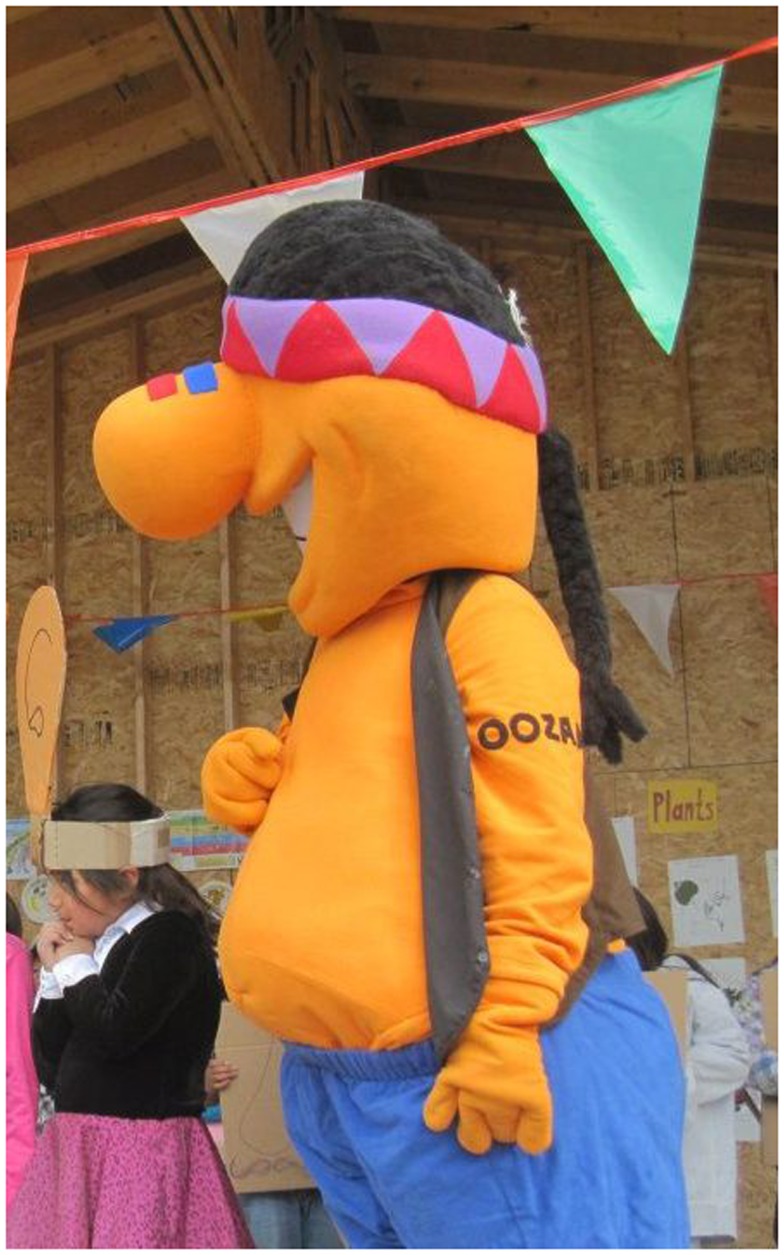
**Chief Sugar Daddy at community health promotion fair**.

Health promotion at community events such as treaty days and Sandy Lake’s Muddy Water Music Festival weekend is another diabetes prevention strategy in SLFN. Pamphlets and healthy recipe cards are made by diabetes prevention program staff to aide in the promotion of healthier lifestyles. Sandy Lake also has a well-known mascot named Chief Sugar Daddy (Figure 3) who attends community events and makes appearances in the school to help with diabetes awareness. The diabetes prevention staff have established a main community activity of focus for each month of the year including, setting up outdoor rinks (February) and making household garden plots (June). The SLHDP staff aim to encourage the people of Sandy Lake to attend the activities held by choosing activities that would interest a large number of people and by using prizes and other incentives. Throughout the year, community members can sign up for various sports tournaments such as baseball, hockey, and broomball, and can also participate in scavenger hunts, weight loss challenges, fitness classes, cooking classes, and walk to work days. Diabetes prevention program staff do not just promote planned exercise, they encourage people to become more active and less sedentary in their everyday activities.

## Discussion

The SLHDP was initiated with four main objectives, all of which have been successfully achieved over the 22 year partnership with the community. (1) The prevalence of diabetes and IGT in Sandy Lake has been previously documented to be 26.1 and 13.6%, respectively (21). (2) Numerous studies have been carried out to identify anthropometric, metabolic, and lifestyle characteristics associated with diabetes and IGT in the community (9, 21, 23, 24). These results increased awareness among community members for the need of programs that promote healthy lifestyles to prevent the onset of diabetes. (3) Ethnographic data collection techniques beginning in 1992 informed the development of culturally appropriate intervention strategies to modify risk factors for diabetes and its complications. Multi-strategy interventions have been implemented in Sandy Lake that combine interventions in the elementary schools and community, involving many settings, organizations, and partners (8–10, 29).

In the last two decades, the SLHDP has had many successes and continues to evolve to align with Sandy Lake’s priorities and needs. The SLHDP staff has worked with the community by soliciting feedback to modify the programs and by hiring community members to lead the programs. The success and duration of this program has been largely due to community participation and ownership. The endorsement of the community was of crucial importance in legitimizing and promoting the project to community members. The joint effort between researchers and community members has allowed the SLHDP to be sustainable and is bringing Sandy Lake closer to diabetes prevention by addressing a number of its most important risk factors. (4) In engaging in a successful community-based participatory research approach, Sandy Lake has provided a model intervention strategy that could be implemented in other First Nations communities. Community-based participatory research has been shown to enhance the relevance of the research to the community by involving key community stakeholders throughout the research process (30). Through the collaborative partnership formed through the SLHDP academics have gained a deeper understanding of the community’s perception of health and illness as it fits within their broader social, cultural, and physical environments. These teachings have helped inform the development and on-going support provided by researchers to the community as the SLHDP has evolved. Completing the circle of reciprocal capacity building, the SLHDP has provided health and research methods training and employment opportunities for a large number of community members.

Sandy Lake First Nation has been successful in establishing relationships between various organizations in the community, which has led to partnerships in community events and programs. By combining efforts and funding, Sandy Lake is able to offer more numerous, higher quality activities directed toward diabetes prevention. It has also resulted in tremendous synergy to capitalize on established funded programs with common goals. Initially, the programs were funded by research grants, although the recognition of the problem highlighted by the research (in Sandy Lake and other communities) has led to permanent funding of a prevention program by the Aboriginal Diabetes Initiative (ADI) of Health Canada (31).

The overarching aim of the SLHDP continues to be the development of strategies to prevent diabetes in Sandy Lake. The key components currently are the diabetes prevention curriculum for grade 3 and 4 students, the weekly diabetes prevention radio show, and community-wide activities developed by the diabetes prevention program staff. Activities on-going in the community are centered around physical activity, nutrition and health education, and include organized sports, cooking classes and a diabetes summer camp.

### Challenges

The SLHDP has faced numerous challenges over its 22 year existence. SLFN is located in an underserviced geographic region where the cost of food is high and the availability of healthy foods is low. Changing lifestyles involves not only educating people so that they can make their own informed decisions, but also changing the environment so that they can more easily make healthy choices is necessary. Providing healthy food for cooking classes, community events, and students is costly and takes over a large portion of the prevention program budget, which in turn diverts funds away from other programing.

In particular, there have been challenges in the school diabetes prevention curriculum implementation and consistency. The curriculum itself has not changed, but some of the original teachers have. There are six different grade 3 and grade 4 classrooms in total, and the teachers do not have the same level of knowledge or enthusiasm toward the curriculum, which is to be taught on top of their normal curriculum.

Ensuring that the programs targeting diabetes prevention are culturally appropriate and sustainable in the community has been of primary concern. To overcome this concern there is continued support from researchers in the form of visits to the community, discussions with staff and on-going presentations to the Chief and Council to determine community needs. In the last two decades, there have been programs initiated by the SLHDP, such as the home visit program, that were not successful as a result of minimal community uptake and the extensive resources that were required to support the program. However, the continued evolution of different strategies developed and initiated by community program staff highlight the importance of community-driven activities, which ultimately will support long-term sustainability.

### Community ownership and program sustainability

Key to the success and sustainability of the SLHDP has been community ownership. The integration of the community as an equal partner is a key principle in the successful sustainment of participatory research. When mutual trust and respect are present, this facilitates ownership of research projects and underpins sustainability (32). Sustainability is an important topic among funders and community partners, and is an indication of program success (32). The likelihood of project sustainability improves through the use of community-based participatory research which embeds an intervention or project within the larger community environment (33). Community-based participatory research is an approach that aspires to create a partnership between investigators and communities that is characterized by mutual respect, shared decision-making, and shared ownership of the project and resulting data as well as a willingness to adapt interventions to increase their relevance to a community (33). Community-based participatory research furthers shared decision-making and decreases the time between interventions and adoption of results by community members (34, 35) and is key in promoting the uptake of programs (33). The most successful programs have been those that the community has adopted and continued even after research funding had stopped. There has been community participation and support for diabetes prevention at many levels, including the Band Council, the Health Authority, and the Education Board, and in different settings, such as in decision-making, conference presentations, and grant authorship. A high degree of community ownership is likely to translate into higher sustainability, as the local community members have a keener sense of what would and would not work.

Sandy Lake First Nation and the Sandy Lake Education Authority have fully supported the diabetes curriculum and the community members have taken ownership of the school-based program despite an end to funding in 2007. The community and teachers continue to be enthusiastic about the curriculum and have supplemented the curriculum with additional after-school activities to promote physical activity. In order to build capacity in the community, SLFN has partnered with Right to Play, which has the dedicated First Nations program PLAY (Promoting Life-Skills in Aboriginal Youth). PLAY is “a multi-faceted program designed to use sport and play activities as a tool to build on the strength of Aboriginal children, youth and their communities, supporting the value of culture and identity.” The PLAY program provides coaches, equipment, and training of community mentors to deliver sport and play-based programs that support physical and social development.

Many facets of the SLHDP have been assessed in pilot and feasibility projects in different communities in Northwestern Ontario (12, 15). The Zhiiwapenewin Akino’maagewin: Teaching to Prevent Diabetes (ZATPD) program was a feasibility study of an integrated multi-institutional diabetes prevention program, based on SLHDP. Similar to SLHDP, ZATPD consisted of school, store, and community components (12, 15). Baseline and follow-up data were collected before and after the 9-month intervention program in seven First Nations communities (15). The seven communities were divided into four sites, two of which were given the intervention, while the other two were used for comparison (15). Results showed a significant change in knowledge among participants in intervention communities (*p* < 0.019) (15). There was also a significant increase in frequency of healthy food acquisition among participants in the intervention communities (*p* < 0.003) (15). However, there were no significant changes in physical activity or BMI in either intervention or comparison groups (15). This study shows that the SLHDP experience provides a model for other First Nations communities in Northwestern Ontario and other remote communities that are also struggling with diabetes. Although each First Nations community is unique, the elements of program implemented in Sandy Lake can be a model for the development of culturally and locally appropriate programs for other remote First Nations communities in the future.

## Conclusion

The SLHDP has been a long-standing community-based intervention with the primary goal of preventing T2DM through a participatory research approach. The initial objectives of the SLHDP were to determine the prevalence of type 2 diabetes and IGT and to identify anthropometric, metabolic, and lifestyle characteristics associated with their development; and to gain an understanding of community perceptions of diabetes and its risk factors though qualitative research. The results of this formative research clarified the need for a community-based intervention; the findings have been essential to the development of culturally appropriate strategies toward diabetes prevention. The most successful components of the intevention have been the school-based diabetes prevention program, the diabetes radio show, the Northern store initiatives, and diabetes prevention programing within the community. The successes of the SLHDP can be attributed to multi-institutional strategies, adaptability, suitability, and community participation and ownership. Aspects of the SLHDP intervention strategy have been assessed through pilot and feasibility research in other First Nations communities in Northwestern Ontario, and the results of this work have indicated that the SLHDP offers a promising model for these communities in developing their own locally appropriate diabetes prevention programs.

## Conflict of Interest Statement

All the authors declare that the research was conducted in the absence of any commercial or financial relationships that could be construed as a potential conflict of interest.
